# The efficacy and safety of JAK inhibitors for alopecia areata: A systematic review and meta-analysis of prospective studies

**DOI:** 10.3389/fphar.2022.950450

**Published:** 2022-08-24

**Authors:** Diqin Yan, Huaying Fan, Min Chen, Lin Xia, Simin Wang, Wenliang Dong, Qian Wang, Suping Niu, Huiying Rao, Liming Chen, Xiaoyan Nie, Yi Fang

**Affiliations:** ^1^ Department of Pharmacy, Peking University People’s Hospital, Beijing, China; ^2^ Department of Pharmacy Administration and Clinical Pharmacy, School of Pharmaceutical Sciences, Peking University, Beijing, China; ^3^ Department of Science and Research, Peking University People’s Hospital, Beijing, China; ^4^ Department of Clinical Pharmacy, Xuzhou Medical University, Xuzhou, China; ^5^ Peking University People’s Hospital, Peking University Hepatology Institute, Beijing Key Laboratory of Hepatitis C and Immunotherapy for Liver Disease, Beijing, China

**Keywords:** alopecia areata, JAK inhibitors, janus kinase inhibitors, tofacitinib, ruxolitinib, baricitinib, meta-analysis, systematic review

## Abstract

**Background:** Due to the lack of comprehensive evidence based on prospective studies, the efficacy and safety of Janus Kinase (JAK) inhibitors (including tofacitinib, ruxolitinib, baricitinib, ritlecitinib and brepocitinib) for alopecia areata (AA) are yet to be proved.

**Methods:** The systematic review and meta-analysis was performed pursuant to the Preferred Reporting Items for Systematic reviews and Meta-Analyses (PRISMA) guideline and registered on PROSPERO (CRD42022303007).

**Results:** Fourteen prospective studies (5 RCTs and 9 non-RCTs), enrolling a total of 1845 patients with AA, were included for quantitative analysis. In RCTs, oral JAK inhibitors resulted in higher good response rate compared with control (RR: 6.86, 95% CI: 2.91–16.16); topical JAK inhibitors did not show any difference compared with control (RR: 1.00, 95% CI: 0.31–3.18). In non-RCTs, the pooled rate of good response to oral, topical and sublingual JAK inhibitors were 63% (95% CI: 44%–80%), 28% (95% CI: 1%–72%) and 11% (95% CI: 1%–29%), respectively. The pooled recurrence rate in patients treated with JAK inhibitors was 54% (95% CI: 39%–69%), mainly due to the withdrawal of JAK inhibitors. In RCTs, no difference was found in the risk of experiencing most kind of adverse events; in non-RCTs, the reported adverse events with high incidence rate were mostly mild and manageable.

**Conclusion:** JAK inhibitors are efficacious and generally well-tolerated in treating AA with oral administration, whereas topical or sublingual administration lacks efficacy. Subgroup analyses indicate that baricitinib, ritlecitinib and brepocitinib seem to have equal efficacy for AA in RCTs; ruxolitinib (vs. tofacitinib) and AA (vs. AT/AU) are associated with better efficacy outcomes in non-RCT. Due to the high recurrence rate after withdrawal of JAK inhibitors, continuous treatment should be considered to maintain efficacy.

**Systematic Review Registration:** PROSPERO: CRD 42022303007

## Introduction

Alopecia areata (AA) is a common, inflammatory, non-scarring type of hair loss. AA presents most commonly as limited patches of hair loss (patchy AA) that can progress to loss of all scalp hairs (alopecia totalis, AT) or all body hairs (alopecia universalis, AU) (Strazzulla et al., 2018). The risk of progression from patchy AA to AT or AU is approximately 5%, and an extensive involvement portends a worse prognosis (Safavi et al., 1995; Tosti et al., 2006). As a common type of alopecia in human, the estimated prevalence of AA is approximately between 0.1% and 0.2%, second only to male and female pattern alopecia (Pratt et al., 2017). And the lifetime incidence risk of AA is approximately 2% (Mirzoyev et al., 2014). The chronic course and frequent relapse of AA can be distressing for patients, even leading to psychosocial disorder and reduction in quality of life. Therefore, the importance should be attached to the treatment of AA.

There are several treatment approaches available for the management of AA, including corticosteroids, minoxidil, topical immunotherapy, cyclosporine, methotrexate, etc (Meah et al., 2020). However, the response of AA patients to these treatments varies widely and adverse events occur frequently especially in systemic medications; few robust and well-designed clinical trials have evaluated and supported these therapies (Lai et al., 2019). Therefore, more effective and less toxic drugs for AA are needed.

As the molecular mechanisms of AA are further defined, targeted therapies including Janus kinase (JAK) inhibitors are considered to be a preferable treatment option. Genome-wide association studies and functional immunological studies have identified that CD8+NKG2D + T cells are the major effectors of AA pathogenesis, which promote the inflammation of hair follicles through interferon-γ (IFN-γ) and interleukin-15 (IL-15) signaling pathways. JAK/signal transducer and activator of transcription (STAT) is in the downstream molecular pathway of IFN-γ and IL-15 receptors (Petukhova et al., 2010; Betz et al., 2015)^,^ (Xing et al., 2014). Therefore, JAK inhibitors can blockade the signaling pathway of AA by inhibiting JAK/STAT activation, leading to the reverse of AA. Among the JAK inhibitors for AA, baricitinib is the first treatment approved for the indication of AA by the Food and Drug Administration (FDA) in 13 June 2022; tofacitinib and ruxolitinib were approved for the treatment of rheumatoid arthritis and other inflammatory disorders; ritlecitinib and brepocitinib are under investigation and not available for routine clinical use. Hence, clinical statistics regarding the efficacy and safety of JAK inhibitors are required to provide a better insight in this new treatment strategy.

Thus, we systematically reviewed the evidence 1) to evaluate the efficacy and safety of JAK inhibitors for AA, 2) to determine the relative efficacy of JAK inhibitors in different administration route (oral vs. topical vs. sublingual administration), and 3) to identify more factors influencing the good response to JAK inhibitors in AA patients.

## Methods

This systematic review and meta-analysis was performed according to the Preferred Reporting Items for Systematic reviews and Meta-Analyses (PRISMA) guideline and registered on PROSPERO (CRD42022303007) (Moher et al., 2009).

### Search strategy

Electronic searches were performed in PubMed, EMBASE and Cochrane library from inception to 17 June 2022, using MeSH or Emtree terms including ‘‘alopecia areata,’’ ‘‘JAK inhibitors,” ‘‘tofacitinib,’’ “ruxolitinib,” ‘‘baricitinib,’’ “ritlecitinib,” and “brepocitinib” and their synonyms. The detailed search strategy for each database is described in the Supplement. We searched the reference lists from retrieved full text articles and previous systematic reviews for further identification of potentially relevant studies. We also searched through PROSPERO for any related systematic reviews.

### Eligibility criteria

Studies were selected based on the following inclusion criteria: 1) studies enrolling human participants with AA/AT/AU; 2) studies in which patients were treated with JAK inhibitors; 3) studies reporting efficacy outcomes including scalp hair regrowth or recurrence rate, or safety outcomes including adverse events; 4) studies of prospective studies including RCTs, clinical trials and prospective cohort studies; 5) studies published in English. Studies were excluded based on the following exclusion criteria: 1) studies enrolling patients without scalp involvement, but only with nails, eyelashes or eyebrows involvement; 2) studies of observational studies, case series, case reports, repeated publications, abstracts, conference presentations, editorials and reviews.

### Data extraction

Two authors independently reviewed the included articles and extracted data on the trial characteristics, baseline characteristics of participants, interventions, comparisons, efficacy and safety outcomes from each trial. Faced with the absence of data, we transformed or estimated measures of variance using the recommendations from the Cochrane Handbook for Systematic Reviews of Interventions (Akl et al., 2019). Any disagreement was resolved by discussion until consensus was reached or by consulting a third author.

The choice of outcomes was based on the usually reported primary and secondary outcomes in clinical trials of AA, AA investigational assessment guidelines (Olsen et al., 2004), and other systematic reviews of AA (Lee et al., 2018; Phan et al., 2019; Phan and Sebaratnam, 2019; Guo et al., 2020). The efficacy outcomes included good response [defined as 50% improvement in Severity of Alopecia Tool (SALT) scores (SALT50)], complete response [defined as 90% improvement in SALT scores (SALT90)], the percent change from baseline in SALT score and recurrence. The safety outcomes included the incidence rates of adverse events.

### Risk of bias assessment

Two authors independently appraised risk of bias of each study using the Cochrane risk of bias tool for RCTs and ROBINS-I (Risk of Bias In Non-randomized Studies-of Interventions) for non-RCTs (including single-arm trials, non-randomized controlled trials and extension periods of RCT) (Higgins et al., 2011) (Sterne et al., 2016). Any disagreement was resolved by discussion until consensus was reached or by consulting a third author.

### Statistical analysis

We conducted meta-analysis of each outcome using the available data for response rates, recurrence rate, and incidence rates of adverse events. All outcomes were reported with associated 95% confidence intervals (CI). Meta-analysis for RCTs and non-RCTs (including single-arm trials, non-randomized controlled trials and extension periods of RCT) were conducted separately. Heterogeneity of the included studies was calculated using Cochran Q statistic (significant at *p* < 0.1) and I^2^ test (significant at I^2^ > 50%). Overall, there was a significant heterogeneity, so a random effects model was used. Preplanned subgroup analysis was conducted according to administration route (oral vs. topical vs. sublingual administration), types of JAK inhibitors (baricitinib vs. ritlecitinib vs. brepocitinib, ruxolitinib vs. tofacitinib), treatment duration (<24 weeks vs. ≥24 weeks) and AA subtype (AA vs. AT/AU). All analyses were performed by the meta package (version 5.1-1) for R (version 4.1.1). *p* value < 0.05 was considered statistically significant.

## Results

### Study selection

**FIGURE 1:**
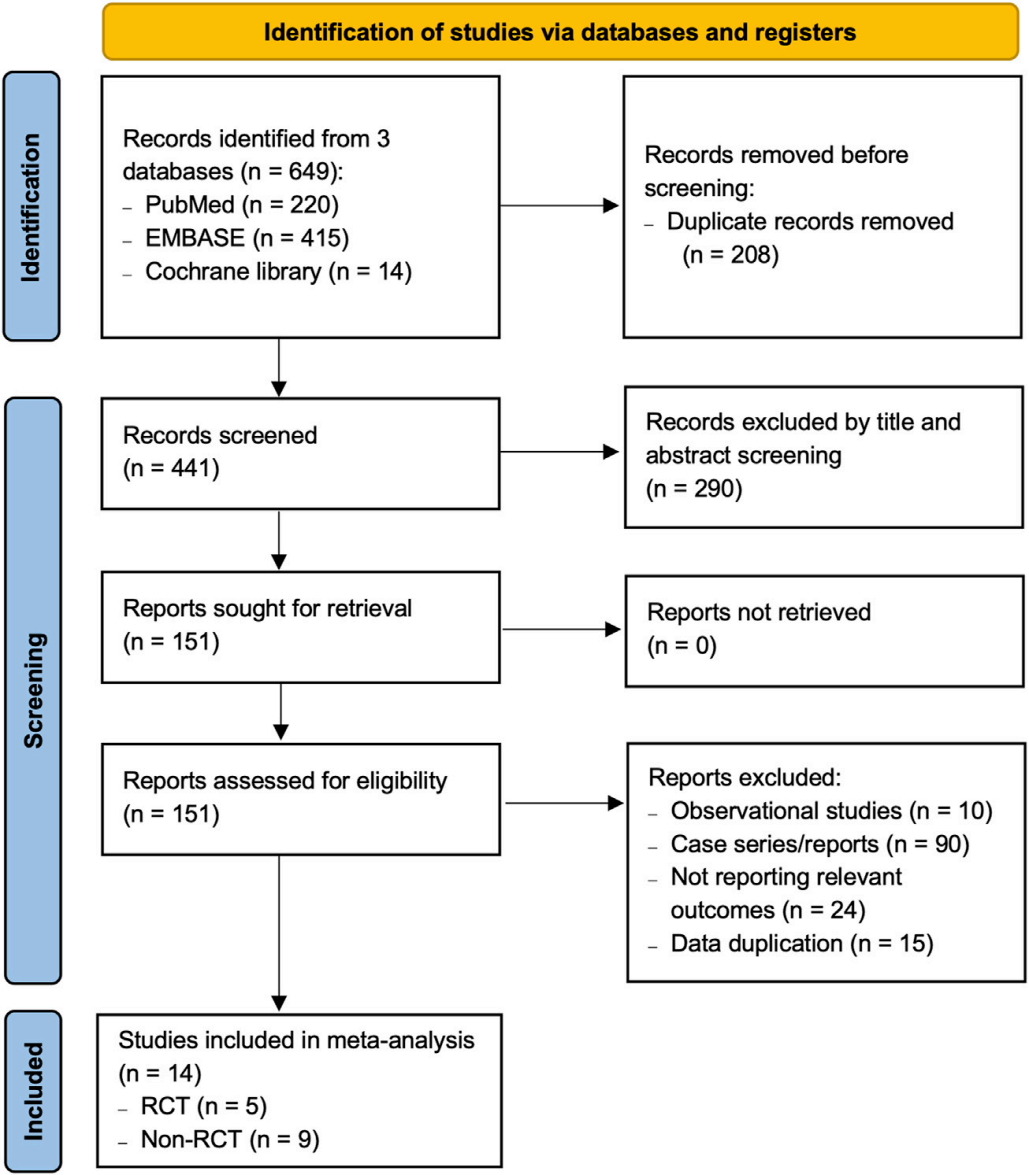
PRISMA flow diagram of record selection process.

Overall, 649 records were identified through three databases. After removing 208 duplicates, we excluded 290 records on the basis of the title and abstract. The remaining 151 potentially relevant reports were reviewed in full text. After detailed evaluation of these reports, 14 studies (5 RCTs and 9 non-RCTs) (Kennedy Crispin et al., 2016; Mackay-Wiggan et al., 2016; Almutairi et al., 2018; Jabbari et al., 2018; Liu et al., 2018; Olsen et al., 2020; King et al., 2021a; AlMarzoug et al., 2021; King et al., 2021b; Lai et al., 2021; Peeva et al., 2021; King et al., 2022), enrolling a total of 1,845 patients, were included for analysis (Figure 1).

### Study characteristics and risk of bias assessment

Characteristics of included studies were described in Table 1. Among the included 5 RCTs, 3 compared oral baricitinib with placebo (King et al., 2021b; King et al., 2022), 1 compared oral ritlecitinib and brepocitinib with placebo (King et al., 2021a), and 1 compared topical ruxolitinib with placebo (Olsen et al., 2020). Among the included 9 non-RCTs, 7 single-arm clinical trials evaluated the efficacy and safety of oral/topical ruxolitinib and oral/topical/sublingual tofacitinib (Kennedy Crispin et al., 2016; Mackay-Wiggan et al., 2016; Jabbari et al., 2018; Liu et al., 2018; Olsen et al., 2020; AlMarzoug et al., 2021; Lai et al., 2021), 1 study of extension periods of RCT investigated the maintenance and withdrawal with oral ritlecitinib and brepocitinib (Peeva et al., 2021), and 1 non-randomized controlled trial compared oral ruxolitinib with oral tofacitinib (Almutairi et al., 2018).

**TABLE 1:** Characteristics of included studies.

Study	Study type	Treatment regimen	Sample size (M: F)	Age (mean/median, SD/range, year)	AA subtype	SALT score (mean/median, SD/range%)	Treatment duration
King et al. (2022) (BRAVE-AA1)	RCT	T1: baricitinib 2 mg QD PO	184 (75:109)	NR (18-60 for male; 18-70 for female)	AA 77 AT 24 AU 83	86.8 (18.0)	36 weeks
		T2: baricitinib 4 mg QD PO	281 (116:165)		AA 133 AT 21 AU 127	85.3 (18.2)	
		C: placebo	189 (80:109)		AA 92 AT 23 AU 74	84.7 (17.8)	
King et al. (2022) (BRAVE-AA2)	RCT	T1: baricitinib 2 mg QD PO	156 (53:103)	NR (18-60 for male; 18-70 for female)	AA 70 AT 16 AU 70	85.6 (18.1)	36 weeks
		T2: baricitinib 4 mg QD PO	234 (90:144)		AA 115 AT 8 AU 111	84.8 (18.1)	
		C: placebo	156 (58:98)		AA 74 AT 16 AU 66	85.0 (17.8)	
King et al. (2021b) (1)	RCT	T1: baricitinib 1 mg QD PO	28 (10:18)	38.6 (11.3)	NR	89.3 (17.7)	36 weeks
		T2: baricitinib 2 mg QD PO	27 (4:23)	42.5 (13.8)		86.1 (19.3)	
		T3: baricitinib 4 mg QD PO	27 (2:25)	42.4 (14.9)		83.4 (17.5)	
		C: placebo	28 (12:16)	40.5 (14.2)		90.0 (15.7)	
King et al. (2021a) (2)	RCT	T1: ritlecitinib 200 mg QD PO for 4 weeks, then 50 mg QD PO for 20 weeks	48 (11:37)	37 (13)	AA 28 AT 7 AU 13	89.4 (15.8)	24 weeks
		T2: brepocitinib 60 mg QD PO for 4 weeks, then 30 mg QD PO for 20 weeks	47 (15:32)	34 (11)	AA 25 AT 8 AU 14	86.4 (18.1)	
		C: placebo	47 (18:29)	38 (14)	AA 27 AT 5 AU 15	88.4 (18.1)	
Olsen et al. (2020) (part B)	RCT	T: topical 1.5% ruxolitinib cream BID	39 (15:24)	44.3 (12.5)	AA 33 AT 6	59.9 (29.4)	24 weeks
		C: topical vehicle cream BID	39 (12:27)	42.4 (12.5)	AA 33 AT 6	59.0 (25.3)	
Olsen et al. (2020) (part A)	CT	Topical 1.5% ruxolitinib cream BID	12 (3:9)	47.6 (10.5)	AA 10	56.2 (21.0)	24 weeks
Peeva et al. (2021)	Extension periods	placebo	22 (NR)	NR	NR	NR	Until subjects lost >30% hair
		placebo	23 (NR)	NR	NR	NR	
Lai et al. (2021)	CT	Sublingual tofacitinib 5 mg BID	18 (4:14)	45.11 (15.28)	AA 5 AT 6 AU 7	86.01 (23.30)	12 weeks
AlMarzoug et al. (2021)	CT	Tofacitinib 5 mg BID PO	65 (28:37)	27.8 (7.81)	AA 17 AT 13 AU 35	76.8 (27.6)	24 weeks
Liu et al. (2018)	CT	Topical tofacitinib 2% ointment BID	10 (6:4)	36.9 (14.2)	NR	77.7 (32.3)	24 weeks
Jabbari et al. (2018)	CT	Tofacitinib 5 mg–10 mg BID PO	12 (4:8)	34.7 (9.59)	AA 7 AT/AU 5	81.3 (22.9)	24–72 weeks
Almutairi et al. (2018)	CT	Ruxolitinib 20 mg BID PO	38 (21:17)	35.5 (13.8)	AA 18 AT 12 AU 8	99.8 (45.50–100)	24 weeks
		Tofacitinib 5 mg BID PO	37 (22:15)	47.4 (16.1)	AA 15 AT 13 AU 9	99.6 (40.37–100)	
Mackay-wiggan et al. (2016)	CT	Ruxolitinib 20 mg BID PO	12 (5:7)	43.67 (14.41)	NR	65.63 (26.01)	12–24 weeks
Kennedy Crispin et al. (2016)	CT	Tofacitinib 5 mg BID PO	66 (35:31)	37 (19–65)	AA 11 AT 6 AU 46	NR	12 weeks

RCT, randomized controlled trial; CT, clinical trial; QD, once a day; BID, twice a day; PO, oral; NR, not report; SD, standard deviation; AA, alopecia areata; AT, alopecia totalis; AU, alopecia universalis; SALT, Severity of Alopecia Tool.

Risk of bias assessment of included studies was described in Table 2. Given the limited number of included studies, we did not remove the studies with high risk of bias.

**TABLE 2:** Risk of bias in (A) included RCTs and (B) included non-RCTs.

Study	Random sequence generation (selection bias)	Allocation concealment (selection bias)		Blinding of participants and personnel (performance bias)	Blinding of outcome assessment (detection bias)	Incomplete outcome data (attrition bias)	Selective reporting (reporting bias)	Other bias
(A)								
King et al. (2022) (BRAVE-AA1)	Low	Low		Low	Low	Low	Low	Low
King et al. (2022) (BRAVE-AA2)	Low	Low		Low	Low	Low	Low	Low
King et al. (2021b) (1)	Unclear	Unclear		Low	Low	Low	Low	Low
King et al. (2021a) (2)	Low	Low		Low	Low	Low	Low	Low
Olsen et al. (2020) (part B)	Low	Low		Low	Unclear	Low	Low	Low
**Study**	**Bias due to confounding**	**Bias in selection of participants into the study**	**Bias in classification of interventions**	**Bias due to deviations from intended interventions**	**Bias due to missing data**	**Bias in measurement of outcomes**	**Bias in selection of the reported result**	**Overall assessment**
(B)								
Peeva et al., 2021	Low	Low	Low	Low	Low	Moderate	Low	Moderate
Olsen et al. (2020) (part A)	Moderate	Low	Low	Low	Low	Moderate	Low	Moderate
Lai et al. (2021)	Serious	Low	Low	Low	Moderate	Moderate	Moderate	Serious
AlMarzoug et al. (2021)	Serious	Low	Low	Low	Serious	Moderate	Moderate	Serious
Liu et al. (2018)	Moderate	Low	Low	Low	Low	Moderate	Moderate	Moderate
Jabbari et al. (2018)	Moderate	Low	Low	Moderate	Low	Moderate	Low	Moderate
Almutairi et al. (2018)	Moderate	Low	Low	Low	Moderate	Moderate	Low	Moderate
Mackay-wiggan et al. (2016)	Moderate	Low	Low	Moderate	Low	Moderate	Low	Moderate
Kennedy Crispin et al. (2016)	Moderate	Low	Low	Low	Moderate	Moderate	Low	Moderate

### Efficacy outcomes

**FIGURE 2:**
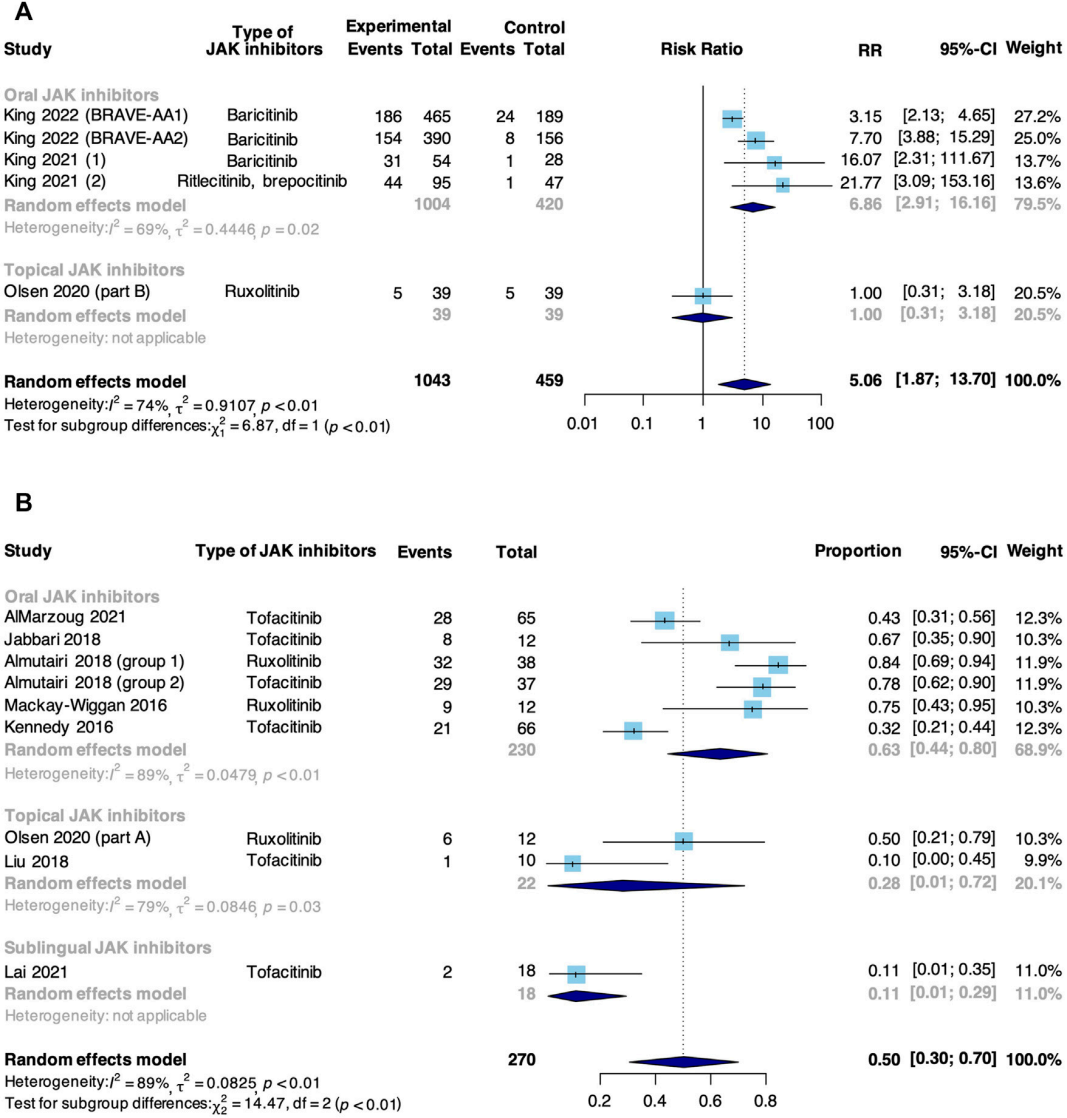
Forest plot of the pooled rate of good response to JAK inhibitors in patients with AA based on **(A)** RCTs and **(B)** non-RCTs.

A good response was defined as the achievement of SALT50. Meta-analysis based on 5 RCTs and 8 non-RCTs evaluated the rate of good response to JAK inhibitors in patients with AA (Figure 2). In RCTs, JAK inhibitors were associated with an increase in the pooled good response rate compared with control (RR: 5.06, 95% CI: 1.87–13.70). Due to high heterogeneity, subgroup analysis was conducted based on the route of administration, and this difference was significant (*p* < 0.01). A significant difference was found in studies where JAK inhibitor was orally administered that the intervention group showed a higher good response rate compared with a controlled group (RR: 6.86, 95% CI: 2.91–16.16), yet such significance was not observed in the study where JAK inhibitor was topically administered (RR: 1.00, 95% CI: 0.31–3.18). In non-RCTs, the pooled rate of good response to JAK inhibitors in AA was 50% (95% CI: 30%–70%). From subgroup analysis, the pooled good response rate in studies where JAK inhibitor was orally administered was 63% (95% CI: 44%–80%), significantly higher than that in studies where participants were treated with topical (28%, 95% CI:1%–72%) and sublingual JAK inhibitors (11%, 95% CI: 1%–29%, *p* < 0.01).

**FIGURE 3:**
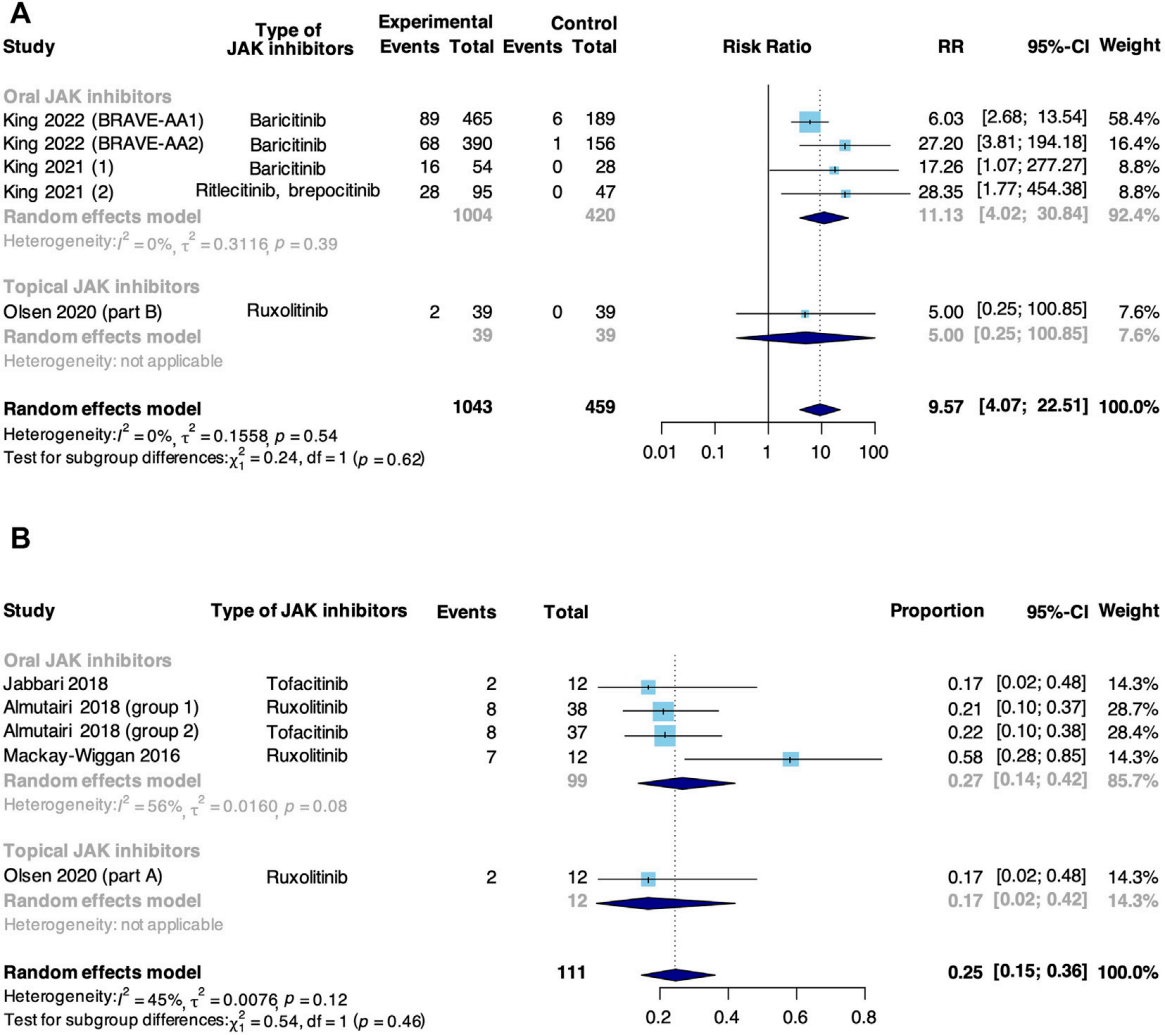
Forest plot of the pooled rate of complete response to JAK inhibitors in patients with AA based on **(A)** RCTs and **(B)** non-RCTs.

A complete response was defined as the achievement of SALT90. Meta-analysis based on 5 RCTs and 4 non-RCTs evaluated the rate of complete response to JAK inhibitors in AA (Figure 3). In RCTs, JAK inhibitors were associated with an increase in the pooled complete response rate compared with control (RR: 9.57, 95% CI: 4.07–22.51). There was no significant difference in subgroup analysis based on the route of administration (*p* = 0.62). However, a significant difference was found in studies where JAK inhibitor was orally administered that the intervention group showed a higher complete response rate compared with a controlled group (RR: 11.13, 95% CI: 4.02–30.84), but not found in the study where JAK inhibitor was topically administered (RR: 5.00, 95% CI: 0.25–100.85). In non-RCTs, the pooled rate of complete response to JAK inhibitors in AA was 25% (95% CI: 15%–36%). From subgroup analysis, the complete response rate in studies where JAK inhibitor was orally administered (27%, 95% CI: 14%–42%) was higher than that in where JAK inhibitor was topically administered (17%, 95% CI: 2%–42%), but the difference was insignificant (*p* = 0.46).

**FIGURE 4:**
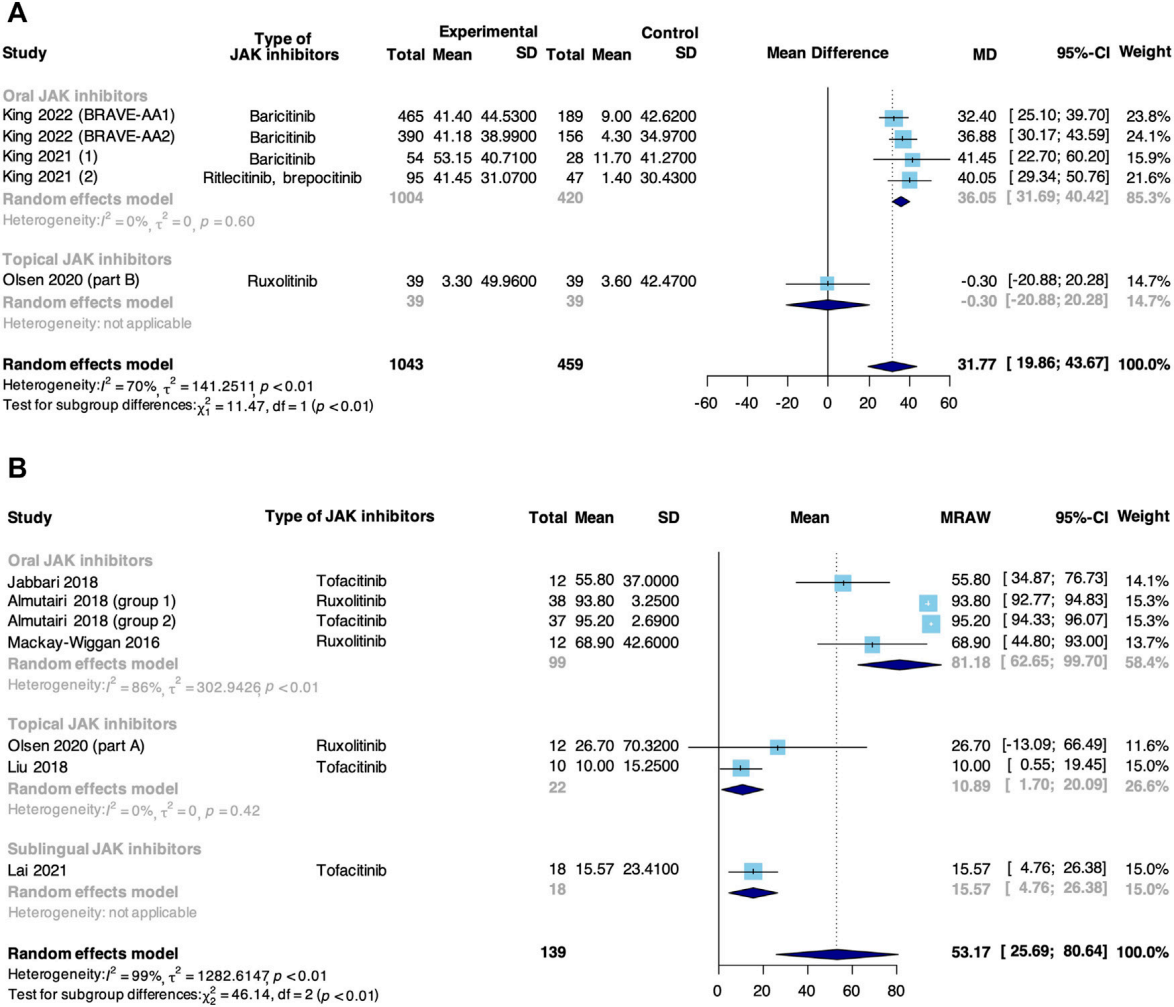
Forest plot of the percent change from baseline in SALT score in patients taking JAK inhibitors for AA based on **(A)** RCTs and **(B)** non-RCTs.

Meta-analysis based on 5 RCTs and 6 non-RCTs evaluated the percent change from baseline in SALT score in patients taking JAK inhibitors for AA (Figure 4). In RCTs, JAK inhibitors were associated with an increase in the percent change from baseline in SALT score compared with control (MD: 31.77, 95% CI: 19.86–43.67). The subgroup analysis revealed that there was a significant difference between oral (MD: 36.05, 95% CI: −31.69–40.42) and topical JAK inhibitors (MD: −0.30, 95% CI: −20.88 to 20.28, *p* < 0.01). In non-RCTs, the pooled percent change from baseline in SALT score was 53.17% (95% CI: 25.69%–80.64%). From subgroup analysis, there was a significant difference among oral (81.18%, 95% CI: 62.65%–99.70%), topical (10.89%, 95% CI: 1.70%–20.09%) and sublingual JAK inhibitors (15.57%, 95% CI: 4.76%–26.38%, *p* < 0.01).

### Subgroup analysis outcomes

Further subgroup analysis was conducted with the good response rate (Table 3). In RCTs, a significant difference was found in terms of administration route (oral vs. topical administration, *p* < 0.01), and no significant difference was observed in terms of types of oral JAK inhibitors (baricitinib vs. ritlecitinib vs. brepocitinib, *p* = 0.55). In non-RCTs, oral administration (vs. topical and sublingual administration, *p* < 0.01), oral ruxolitinib (vs. oral tofacitinib, *p* = 0.02), topical ruxolitinib (vs. topical tofacitinib, *p* = 0.03) and AA (vs. AT/AU, *p* = 0.04) were associated with better response outcomes, with statistical significance; no significant difference was found in terms of treatment duration (≥24 weeks vs. <24 weeks, *p* = 0.28).

**TABLE 3:** Subgroup analysis based on (A) RCTs and (B) non-RCTs.

Variable	No of participants (No of trials)	RR (95% CI)	Heterogeneity		Test for subgroup differences
			I^2^ (%)	*p*-value	*p*-value
**(A)**					
Administration route					
Oral JAK inhibitors	1424 (4)	6.86 (2.91; 16.16)	69	0.02	<0.01
Topical JAK inhibitors	78 (1)	1.00 (0.31; 3.18)	NA	NA	
Types of oral JAK inhibitors					
Baricitinib	1282 (3)	5.58 (2.42; 12.87)	71	0.03	0.55
Ritlecitinib	72 (1)	9.50 (1.35; 66.79)	NA	NA	
Brepocitinib	70 (1)	25.23 (1.61; 396.61)	NA	NA	

Variable	No of participants (No of trials)	RR (95% CI)	Heterogeneity		Test for subgroup differences
			I^2^ (%)	*p*-value	*p*-value
**(B)**					
Administration route					
Oral JAK inhibitors	230 (6)	0.63 (0.44; 0.80)	89	<0.01	<0.01
Topical JAK inhibitors	22 (2)	0.28 (0.01; 0.72)	79	0.03	
Sublingual JAK inhibitors	18 (1)	0.11 (0.01; 0.35)	NA	NA	
Types of oral JAK inhibitors					
Tofacitinib	180 (4)	0.54 (0.34; 0.73)	85	<0.01	0.02
Ruxolitinib	50 (2)	0.82 (0.69; 0.90)	0	0.47	
Types of topical JAK inhibitors					
Tofacitinib	10 (1)	0.10 (0.00; 0.35)	NA	NA	0.03
Ruxolitinib	12 (1)	0.50 (0.23; 0.77)	NA	NA	
Duration of treatment					
≥24 weeks	174 (5)	0.50 (0.30; 0.70)	82	<0.01	0.28
<24 weeks	96 (3)	0.35 (0.12; 0.69)	81	<0.01	
AA subtype					
AA	68 (4)	0.80 (0.65; 0.89)	NA	NA	0.04
AT/AU	147 (4)	0.47 (0.22; 0.74)	NA	NA	

### Recurrence outcomes

**FIGURE 5:**
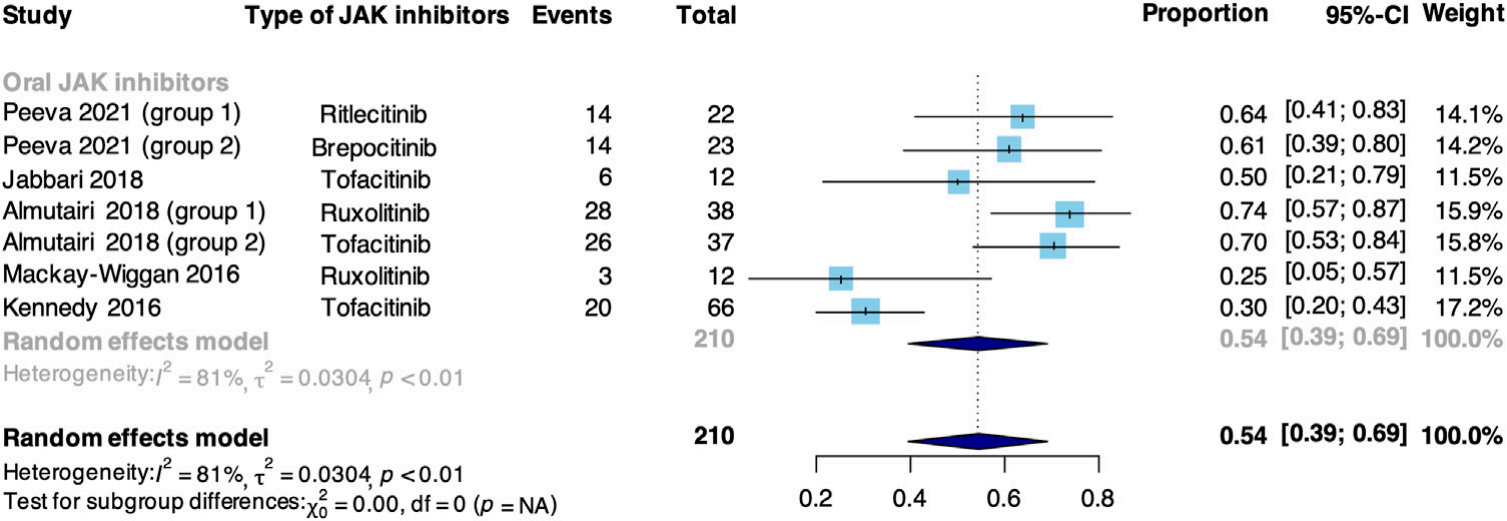
Forest plot of recurrence rate in patients treated with JAK inhibitors based on non-RCTs.

Meta-analysis based on 5 non-RCTs evaluated the recurrence rate in patients treated with JAK inhibitors (Figure 5). The pooled recurrence rate was 54% (95% CI: 39%–69%). The main cause of recurrence was the withdrawal of JAK inhibitors.

### Safety outcomes

Meta-analysis based on 5 RCTs and 5 non-RCTs evaluated the safety of JAK inhibitors in patients with AA (Table 4). The types and reporting of adverse events varied across different studies. In RCTs, there was no significant difference between JAK inhibitors and placebo in the risk of experiencing treatment-emergent adverse event (TEAE, RR: 1.05, 95% CI: 0.96–1.14), serious AE (RR: 1.61, 95% CI: 0.70–3.68), upper respiratory tract infection (URTI, RR: 1.12, 95% CI: 0.76–1.67), headache (RR: 1.13, 95% CI: 0.72–1.77) and nasopharyngitis (RR: 1.00, 95% CI: 0.64–1.58). Acne was more common with baricitinib than with placebo (RR: 3.48, 95% CI: 1.55 to 7.82, *p* < 0.01). In non-RCTs, the highest risk was observed for URTI (37.05%), followed by diarrhea (19.65%), acne (9.31%), urinary tract infection (UTI, 6.98%), headache (6.33%) and folliculitis (4.48%).

**TABLE 4:** Adverse events and incidence rate in (A) RCTs and (B) non-RCTs.

Adverse effects	No of participants (No of trials)	Effect size		Heterogeneity	
		RR (95% CI)	*p*-value	I^2^ (%)	*p*-value
**(A)**					
TEAE	1502 (5)	1.05 (0.96; 1.14)	0.32	0	0.42
Serious AE	1502 (5)	1.61 (0.70; 3.68)	0.26	0	0.96
URTI	1424 (4)	1.12 (0.76; 1.67)	0.57	0	0.88
Acne	1424 (4)	3.48 (1.55; 7.82)	<0.01	0	0.63
Headache	1342 (3)	1.13 (0.72; 1.77)	0.61	0	0.83
Nasopharyngitis	1342 (3)	1.00 (0.64; 1.58)	0.99	0	0.87

Adverse effects	No of participants (No of trials)	Incidence rate, % (95% CI, %)	Heterogeneity	
			I^2^ (%)	*p*-value
**(B)**				
URTI	165 (4)	37.05 (7.39; 81.28)	90.2	<0.01
Diarrhea	165 (4)	19.65 (0.00; 43.24)	85.6	<0.01
Acne	90 (3)	9.31 (3.39; 15.24)	14.0	0.31
UTI	153 (3)	6.98 (0.23; 13.73)	55.4	0.11
Headache	153 (3)	6.33 (2.48; 10.19)	0	0.84
Folliculitis	151 (3)	4.48 (1.19; 7.76)	0	0.50

TEAE, treatment-emergent adverse event; URTI, upper respiratory tract infection; UTI, urinary tract infection.

## Discussion

### Main findings

In this systematic review and meta-analysis, 14 prospective studies (5 RCTs and 9 non-RCTs), including a total of 1845 participants with AA, were enrolled for syntheses. Overall, our results confirm that oral JAK inhibitors can be a promising option for the treatment of AA, which is corroborated as the JAK inhibitor was first approved for treatment of AA by FDA.

The efficacy outcomes demonstrated, based on both RCTs and non-RCTs, that oral JAK inhibitors could induce hair regrowth significantly in terms of all efficacy outcomes (including good response rate, complete response rate and the percent change from baseline in SALT score). On the contrary, there was no significant difference in efficacy outcomes between topical JAK inhibitors and placebo control based on RCTs; topical and sublingual JAK inhibitors induced minimal hair regrowth in terms of all efficacy outcomes based on non-RCTs, and the improvement was too little to be clinical meaningful or to be distinguished from the spontaneous remission and placebo effect. Our results were in line with previous study. Olsen et al. reported potential efficacy of topical ruxolitinib in part A (an open-label and single-arm clinical trial), but there was no significant difference in hair regrowth between topical ruxolitinib group and control group in part B (an RCT) (Olsen et al., 2020). The different findings between the two parts could be explained by the fact that the spontaneous remission of AA and placebo effect were mistakenly attributed to topical ruxolitinib in non-RCT, whereas the placebo control eliminated such biases in RCT, thus revealing the true response to topical ruxolitinib. Therefore, the finding of part B that topical ruxolitinib did not have a significant effect for AA was more convincing.

Cytokine receptors are paired with different JAKs [including JAK1, JAK2, JAK3, and tyrosine kinase 2 (TYK2)], which are activated upon cytokine binding. JAK2 mediates IFN-γ receptor signaling, JAK3 mediates γc cytokine receptor signaling, TYK2 mediates IFN-α/β receptor signaling, and JAK1 mediates these three cytokine receptor signaling pathways (O'Shea et al., 2013). Among 5 types of JAK inhibitors included in this study, tofacitinib is a JAK1/3 inhibitor, ruxolitinib and baricitinib are JAK1/2 inhibitors, ritlecitinib is a JAK3 selective inhibitor, and brepocitinib is a JAK1/TYK2 inhibitor (Xing et al., 2014; King et al., 2021a; King et al., 2021b). According to the results of subgroup analysis based on types of JAK inhibitors, there was no significant difference observed among baricitinib, ritlecitinib and brepocitinib in RCTs. In non-RCTs, ruxolitinib was associated with better response outcomes, compared with tofacitinib. But the results of subgroup analysis need further verification because of inadequate reporting data and limited number of participants. Additionally, due to the limited types of selective JAK inhibitors included, it is hard to identify the relative contribution of JAK1, JAK2, JAK3, and TYK2 inhibition to the therapeutic effect on AA. However, some other studies demonstrated that IFN-γ (via JAK1/2) and γc cytokine (*via* JAK1/3) signaling pathways play key roles in AA pathogenesis, but the role of IFN-α/β (via JAK1/TYK2) in AA remains undefined. Besides, JAK2 is essential for the function of hematopoiesis-related cytokines, including erythropoietin, thrombopoietin, growth hormone, and granulocyte-macrophage colony-stimulating factor (GM-CSF) (Neubauer et al., 1998). Hence, the blockade of JAK2 may lead to potential side effect, including anemia, thrombocytopenia, and neutropenia. Dai et al. found that JAK1 and JAK3 selective inhibitors robustly induced hair regrowth and decreased AA-associated inflammation, whereas JAK2 selective inhibitors failed to restore hair growth in C3H/HeJ mice with AA (Dai et al., 2021). Furthermore, unlike JAK1, which is broadly expressed in many tissues, the expression of JAK3 is mainly restricted to lymphocytes (Elwood et al., 2017), so that the inhibition of JAK3 signaling may be sufficient to reverse AA. Overall, JAK1 or JAK3 (especially JAK3) selective inhibitors may be a wise choice for AA, for they are theoretically related to less hematologic toxicity and more precise efficacy.

There was a contradiction among the results of subgroup analysis, recurrence and safety assessment. The results of subgroup analysis based on treatment duration showed that no significant difference was found between the treatment duration ≥24 weeks and <24 weeks. Paradoxically, the recurrence assessment indicated that approximately a half of patients treated with JAK inhibitors experienced disease relapse, and the main cause of recurrence was the withdrawal of JAK inhibitors. Peeva et al. reported 16 of 29 (55%) relapsed patients receiving re-treatment with JAK inhibitors achieved primary endpoint again (Peeva et al., 2021). Therefore, several studies suggested that to maintain hair regrowth, continuous treatment should be considered in patients who are tolerated and responsive to JAK inhibitors (Kennedy Crispin et al., 2016; Almutairi et al., 2018; Peeva et al., 2021). Unfortunately, to our knowledge there is no consensus on the optimal interval or duration of maintenance treatment. In addition, although the safety assessment reflects that JAK inhibitors are safe, the long-term safety is still in doubt because of limited experience with JAK inhibitors for the treatment of AA. According to the molecular mechanism of JAK inhibitors, immunosuppression will increase the risk of infection (O'Shea et al., 2004). Some studies on the safety of JAK inhibitors in rheumatic disease indicated that JAK inhibitors were associated with a decrease in neutrophil count and an increased risk of viral infection, particularly herpes zoster (Winthrop, 2017; Harigai, 2019). Based upon the above, the acceptable benefit-risk ratio can be obtained by early identifying strong responders, slow responders and non-responders to JAK inhibitors and then respectively applying optimal courses of treatment. AA disease activity index (ALADIN) score and AA responsiveness to JAK/STAT inhibitors (AARSIN) score were developed to effectively stratify AA patients based on disease phenotype, which may be useful as predictive biomarkers for response to JAK inhibitors (Xing et al., 2014; Kennedy Crispin et al., 2016; Mackay-Wiggan et al., 2016; Jabbari et al., 2018). Kennedy et al. stratified AA patients by AARSIN score, and 2 patients in the slow responder group who continued tofacitinib for an additional 3 months achieved SALT50, which demonstrated that longer treatment course or more potent JAK inhibitors could be beneficial to slow responders (Kennedy Crispin et al., 2016).

### Strengths and weaknesses

Different from the previous systematic reviews (Phan and Sebaratnam, 2019; Guo et al., 2020), which were mainly based on observational studies of low-quality, we included multiple varieties of JAK inhibitors evaluated in prospective studies (including RCTs, single-arm clinical trials, non-randomized controlled trials and extension periods of RCT) so that the more comprehensive evidence on the efficacy and safety of JAK inhibitors were obtained. To appraise the risk of bias of each study, we used the Cochrane risk of bias tool for RCTs and ROBINS-I for non-RCTs separately (Higgins et al., 2011; Sterne et al., 2016). Considering the differences of methodology and quality between RCTs and non-RCTs, we performed meta-analysis for them respectively.

Due to inadequate data reporting, we did not include several relevant trials in meta-analysis (King et al., 2021c; Ko et al., 2021; Senna et al., 2021). The publication language was restricted to English so that some relevant trials could have been missed. Although we included updated information based on prospective studies, better evidence could have been provided if there were more robust and well-designed RCTs comparing JAK inhibitors with negative or positive control. One of the major limitations of this review was the high heterogeneity of the studies, which could result from the inclusion of three routes of administration. For this reason, a random effects model was used and subgroup analyses were conducted to reduce heterogeneity.

## Conclusion

JAK inhibitors are efficacious and generally well-tolerated in treating AA with oral administration, whereas topical or sublingual administration lacks efficacy. Subgroup analyses indicate that baricitinib, ritlecitinib and brepocitinib seem to have equal efficacy for AA in RCTs; ruxolitinib (vs. tofacitinib) and AA (vs. AT/AU) are associated with better efficacy outcomes in non-RCT. Given the high recurrence rate after withdrawal of JAK inhibitors, continuous treatment should be considered to maintain efficacy.

## Data Availability

The original contributions presented in the study are included in the article/Supplementary Material, further inquiries can be directed to the corresponding authors.
