# Supplementary material for: The efficacy and safety of JAK inhibitors for alopecia areata: A systematic review and meta-analysis of prospective studies
Source: Front Pharmacol. 2022 Aug 24;13:950450. doi: 10.3389/fphar.2022.950450 (PMC9449963; doi:10.3389/fphar.2022.950450)
Supplement: Supplementary file 1 [file Table1.DOCX]

Supplemental file 1. Search strategy

(1) Search Strategy for PubMed

| ID | Search |
| --- | --- |
| #1 | ((("Alopecia Areata"[Mesh]) OR (Alopecia Circumscripta)) OR ((((("Alopecia universalis" [Supplementary Concept]) OR (Atrichia, Generalized)) OR (Generalized Atrichia)) OR (Alopecia universalis congenita)) OR (ALUNC Alopecia universalis congenitalis))) OR (alopecia totali) |
| #2 | ((((((((("Janus Kinase Inhibitors"[Mesh]) OR (Inhibitors, Janus Kinase)) OR (Kinase Inhibitors, Janus)) OR (JAK Inhibitors)) OR (Inhibitors, JAK)) OR (Janus Kinase Inhibitor)) OR (Inhibitor, Janus Kinase)) OR (Kinase Inhibitor, Janus)) OR (JAK Inhibitor)) OR (Inhibitor, JAK) |
| #3 | (((((((("tofacitinib" [Supplementary Concept]) OR (tasocitinib)) OR (tofacitinib citrate)) OR (Xeljanz)) OR (CP 690,550)) OR (CP690550)) OR (CP-690550)) OR (CP 690550)) OR (CP-690,550) |
| #4 | (((((((((((((((("ruxolitinib" [Supplementary Concept]) OR (3R)-3-cyclopentyl-3-(4-(7H-pyrrolo(2,3-d)pyrimidin-4-yl)pyrazol-1-yl)propanenitrile)) OR (ruxolitinib phosphate)) OR (ruxolitinib monophosphate)) OR (INCB-18424 phosphate)) OR (Jakavi)) OR (INCB018424 phosphate)) OR (INCB-018424 salt)) OR (Jakafi)) OR (ruxolitinib (as phosphate))) OR (INCB-018424 phosphate)) OR (INCB-018424)) OR (INC-424)) OR (INCB-18424)) OR (INC424)) OR (INCB018424)) OR (INCA24) |
| #5 | (((((((((("baricitinib" [Supplementary Concept]) OR (3-azetidineacetonitrile, 1-(ethylsulfonyl)-3-(4-(7H-pyrrolo(2,3-d)pyrimidin-4-yl)-1H-pyrazol-1-yl)-)) OR (baricitinib phosphate)) OR (3-azetidineacetonitrile, 1-(ethylsulfonyl)-3-(4-(7H-pyrrolo(2,3-d)pyrimidin-4-yl)-1H-pyrazol-1-yl)-, phosphate (1:1))) OR (baricitinib phosphate salt)) OR (INCB-28050)) OR (Olumiant)) OR (INCB028050)) OR (INCB-028050)) OR (LY3009104)) OR (LY-3009104) |
| #6 | (ritlecitinib) OR (brepocitinib) |
| #7 | #2 OR #3 OR #4 OR #5 OR #6 |
| #8 | #1 AND #7 |

(2) Search Strategy for EMBASE

| ID | Search |
| --- | --- |
| #1 | 'alopecia areata'/exp |
| #2 | 'allopecia areata maligna':ab,ti OR 'alopecia circumscripta':ab,ti OR 'area celsi':ab,ti OR 'areate alopecia':ab,ti |
| #3 | 'alopecia universalis'/exp |
| #4 | 'atrichia, generalized':ab,ti OR 'generalized atrichia':ab,ti OR 'alopecia universalis congenita':ab,ti OR 'alunc alopecia universalis congenitalis':ab,ti OR 'alopecia universalis':ab,ti |
| #5 | 'alopecia totalis':ab,ti |
| #6 | #1 OR #2 OR #3 OR #4 OR #5 |
| #7 | 'janus kinase inhibitor'/exp |
| #8 | 'inhibitors, janus kinase':ab,ti OR 'kinase inhibitors, janus':ab,ti OR 'jak inhibitors':ab,ti OR 'inhibitors, jak':ab,ti OR 'janus kinase inhibitors':ab,ti OR 'inhibitor, janus kinase':ab,ti OR 'kinase inhibitor, janus':ab,ti OR 'jak inhibitor':ab,ti OR 'inhibitor, jak':ab,ti OR 'janus tyrosine kinase inhibitor':ab,ti |
|  | 'tofacitinib'/exp |
|  | 'cp 690 550':ab,ti OR 'cp 690, 550':ab,ti OR 'cp 690550':ab,ti OR 'cp 690550 10':ab,ti OR 'cp 690550-10':ab,ti OR 'cp690 550':ab,ti OR 'cp690, 550':ab,ti OR cp690550:ab,ti OR 'cp690550 10':ab,ti OR tasocitinib:ab,ti OR 'tasocitinib citrate':ab,ti OR 'tofacitinib citrate':ab,ti OR xeljanz:ab,ti OR 'xeljanz xr':ab,ti |
| #9 | 'ruxolitinib'/exp |
| #10 | '3 [4 (7h pyrrolo [2, 3 d] pyrimidin 4 yl) 1h pyrazol 1 yl] 3 cyclopentylpropanenitrile':ab,ti OR 'inc 424':ab,ti OR inc424:ab,ti OR 'incb 018424':ab,ti OR 'incb 18424':ab,ti OR 'incb 424':ab,ti OR incb018424:ab,ti OR incb18424:ab,ti OR incb424:ab,ti OR jakafi:ab,ti OR jakavi:ab,ti OR 'ruxolitinib maleate':ab,ti OR 'ruxolitinib phosphate':ab,ti |
| #11 | 'baricitinib'/exp |
| #12 | 'incb 028050':ab,ti OR 'incb 28050':ab,ti OR incb028050:ab,ti OR incb28050:ab,ti OR 'ly 3009104':ab,ti OR ly3009104:ab,ti OR olumiant:ab,ti |
| #13 | 'ritlecitinib':ab,ti OR 'brepocitinib':ab,ti |
| #14 | #7 OR #8 OR #9 OR #10 OR #11 OR #12 OR #13 |
| #15 | #6 AND #14 |

(3) Search Strategy for Cochrane

| ID | Search |
| --- | --- |
| #1 | MeSH descriptor: [Alopecia Areata] explode all trees |
| #2 | (Alopecia universalis):ti,ab,kw (Word variations have been searched) |
| #3 | (alopecia totalis):ti,ab,kw (Word variations have been searched) |
| #4 | #1 OR #2 OR #3 |
| #5 | MeSH descriptor: [Janus Kinase Inhibitors] explode all trees |
| #6 | (Inhibitor, Janus Kinase):ti,ab,kw OR (Kinase Inhibitors, Janus):ti,ab,kw OR (Inhibitors, Janus Kinase):ti,ab,kw OR (Inhibitor, JAK):ti,ab,kw OR (Janus Kinase Inhibitor):ti,ab,kw (Word variations have been searched) |
| #7 | (Kinase Inhibitor, Janus):ti,ab,kw OR (JAK Inhibitors):ti,ab,kw OR (Inhibitors, JAK):ti,ab,kw OR (JAK Inhibitor):ti,ab,kw (Word variations have been searched) |
| #8 | (tofacitinib):ti,ab,kw OR (tasocitinib):ti,ab,kw OR (tofacitinib citrate):ti,ab,kw OR (Xeljanz):ti,ab,kw OR (CP 690,550):ti,ab,kw (Word variations have been searched) |
| #9 | (CP690550):ti,ab,kw OR (CP-690550):ti,ab,kw OR (CP 690550):ti,ab,kw OR (CP-690,550):ti,ab,kw (Word variations have been searched) |
| #10 | (ruxolitinib):ti,ab,kw OR (Jakavi):ti,ab,kw OR (Jakafi):ti,ab,kw OR (INCB-018424):ti,ab,kw OR (INC-424):ti,ab,kw (Word variations have been searched) |
| #11 | (INCB-18424):ti,ab,kw OR (INC424):ti,ab,kw OR (INCB018424):ti,ab,kw OR (INCA24):ti,ab,kw (Word variations have been searched) |
| #12 | (baricitinib):ti,ab,kw OR (INCB-28050):ti,ab,kw OR (Olumiant):ti,ab,kw OR (INCB028050):ti,ab,kw OR (INCB-028050):ti,ab,kw (Word variations have been searched) |
| #13 | (LY3009104):ti,ab,kw OR (LY-3009104):ti,ab,kw (Word variations have been searched) |
| #14 | (ritlecitinib):ti,ab,kw OR (brepocitinib):ti,ab,kw (Word variations have been searched) |
| #15 | #5 OR #6 OR #7 OR #8 OR #9 OR #10 OR #11 OR #12 OR #13 OR #14 |
| #16 | #4 AND #15 |
